# “I” and “Me”: The Self in the Context of Consciousness

**DOI:** 10.3389/fpsyg.2018.01656

**Published:** 2018-09-04

**Authors:** Mateusz Woźniak

**Affiliations:** Cognition and Philosophy Lab, Department of Philosophy, Monash University, Melbourne, VIC, Australia

**Keywords:** self, consciousness, self-consciousness, sense of self, self-as-subject, self-as-object, predictive coding, IIT

## Abstract

James (1890) distinguished two understandings of the self, the self as “Me” and the self as “I”. This distinction has recently regained popularity in cognitive science, especially in the context of experimental studies on the underpinnings of the phenomenal self. The goal of this paper is to take a step back from cognitive science and attempt to precisely distinguish between “Me” and “I” in the context of consciousness. This distinction was originally based on the idea that the former (“Me”) corresponds to the self as an object of experience (self as object), while the latter (“I”) reflects the self as a subject of experience (self as subject). I will argue that in most of the cases (arguably all) this distinction maps onto the distinction between the phenomenal self (reflecting self-related content of consciousness) and the metaphysical self (representing the problem of subjectivity of all conscious experience), and as such these two issues should be investigated separately using fundamentally different methodologies. Moreover, by referring to Metzinger’s (2018) theory of phenomenal self-models, I will argue that what is usually investigated as the phenomenal-“I” [following understanding of self-as-subject introduced by Wittgenstein (1958)] can be interpreted as object, rather than subject of experience, and as such can be understood as an element of the hierarchical structure of the phenomenal self-model. This understanding relates to recent predictive coding and free energy theories of the self and bodily self discussed in cognitive neuroscience and philosophy.

## Introduction

Almost 130 years ago, James (1890) introduced the distinction between “Me” and “I” (see **Table 1** for illustrative quotes) to the debate about the self. The former term refers to understanding of the self as an object of experience, while the latter to the self as a subject of experience^1^. This distinction, in different forms, has recently regained popularity in cognitive science (e.g., Christoff et al., 2011; Liang, 2014; Sui and Gu, 2017; Truong and Todd, 2017) and provides a useful tool for clarifying what one means when one speaks about the self. However, its exact meaning varies in cognitive science, especially in regard to what one understands as the self as subject, or “I.”

**Table 1:** Quotes from James (1890) illustrating the distinction between self-as-object (“Me”) and self-as-subject (“I”) and a quote from Wittgenstein (1958) illustrating his distinction between the use of “I” as object and as subject.

	Description and illustrative quote
James, 1890	James (1890) **on the distinction between “me” and “not-me,” and their relation to “I” (the Thinker):** “We may sum up by saying that personality implies the incessant presence of two elements, and objective person, known by a passing subjective Thought and recognized as continuing in time. Hereafter let us see the words ME and I for the empirical person and the judging Thought.,” p. 371 “(…) it would follow that all that is experienced is, strictly considered, objective; that this Objective falls asunder into two contrasted parts, one realized as ‘Self,’ the other as ‘not-Self;’ and that over and above these parts there is nothing save the fact that they are known, the fact of the stream of thought being there as the indispensable subjective condition of their being experienced at all. But this condition of the experience is not one of the things experienced at the moment; this knowing is not immediately known. It is only known in subsequent reflection. (…) Each ‘section’ of the stream would then be a bit of sciousness or knowledge of this sort, including and contemplating its ‘me’ and its ‘not-me’ as objects which work out their drama together, but not yet including or contemplating its own subjective being. (…) The sciousness in question would be the Thinker, and the existence of this thinker would be given to us rather as a logical postulate than as that direct inner perception” p. 304
James, 1890	James (1890) **on “I” (referred to as the Thinker) as a metaphysical issue:** “But who the Thinker would be, or how many distinct Thinkers we ought to suppose in the universe, would all be subjects for an ulterior metaphysical inquiry,” p. 304
Wittgenstein, 1958	Wittgenstein’s (1958) **distinction between the use of “I” as subject and as object:** “There are two different cases in the use of the word “I” (or “my”) which I might call “the use as object” and “the use as subject.” Examples of the first kind are these: “My arm is broken,” “I have grown six inches,” “I have a bump on my forehead,” “The wind blows my hair about.” Examples of the second kind are: “I see so-and-so,” “I hear so-and-so,” “I try to lift my arm,” “I think it will rain,” “I have toothache.” (…) It is possible that, say in an accident, I should feel a pain in my arm, see a broken arm at my side, and think it is mine, when really it is my neighbor’s. And I could, looking into a mirror, mistake a bump on his forehead for one on mine. On the other hand, there is no question of recognizing a person when I say I have a toothache. To ask “are you sure it’s you who have pain?” would be nonsensical.”, pp. 66–67


**FIGURE 1:**
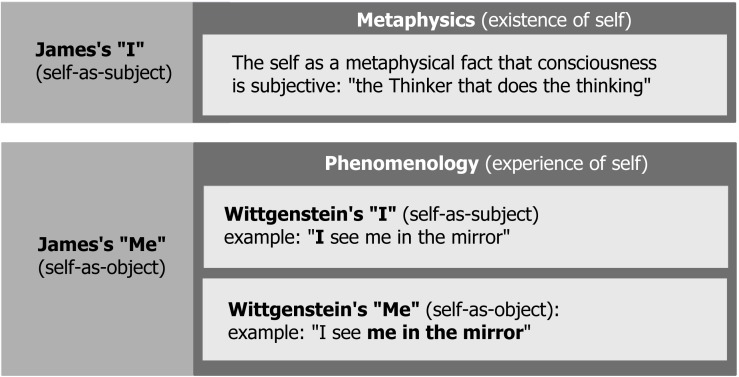
An illustration of James (1890) and Wittgenstein’s (1958) distinctions between self-as-object (“Me”) and self-as-subject (“I”). In the original formulation, James’ (1890) “Me” includes also physical objects and people (material and social “Me”) – they were not included in the picture, because they are not directly related to consciousness.

The goal of this paper is to take a step back from cognitive science and take a closer look at the conceptual distinction between “Me” and “I” in the context of consciousness. I will suggest, following James (1890) and in opposition to the tradition started by Wittgenstein (1958), that in this context “Me” (i.e., the self as object) reflects the phenomenology of selfhood, and corresponds to what is also known as sense of self, self-consciousness, or phenomenal selfhood (e.g., Blanke and Metzinger, 2009; Blanke, 2012; Dainton, 2016). On the other hand, the ultimate meaning of “I” (i.e., the self as subject) is rooted in metaphysics of subjectivity, and refers to the question: why is all conscious experience subjective and who/what is the subject of conscious experience? I will argue that these two theoretical problems, i.e., phenomenology of selfhood and metaphysics of subjectivity, are in principle independent issues and should not be confused. However, cognitive science usually follows the Wittgensteinian tradition^2^ by understanding the self-as-subject, or “I,” as a phenomenological, rather than metaphysical problem [**Figure 1** illustrates the difference between James (1890) and Wittgenstein’s (1958) approach to the self]. By following Metzinger’s (2003, 2010) framework of phenomenal self-models, and in agreement with a reductionist approach to the phenomenal “I”^3^ (Prinz, 2012), I will argue that what is typically investigated in cognitive science as the phenomenal “I” [or the Wittgenstein’s (1958) self-as-subject] can be understood as just a higher-order component of the self-model reflecting the phenomenal “Me.” **Table 2** presents some of crucial claims of the theory of self-models, together with concise references to other theories of the self-as-object discussed in this paper.

**Table 2:** Examples of theories of the self-as-object (“Me”) in the context of consciousness, as theories of the phenomenal self, with representative quotes illustrating each position.

	Description and illustrative quote
**Phenomenal self**	**The self understood as a conscious experience of being “me” (or “I”), also investigated as phenomenal selfhood, self-consciousness, or sense of self.**
Metzinger, 2010	**Minimal phenomenal selfhood (MPS) as a conscious experience (with a specific content) of being a self:** “What does exist is an intermittent process, the experience of being a self, as well as the diverse and constantly changing contents of self-consciousness. This is what philosophers mean when they talk about the ‘phenomenal self’: The way you appear to yourself, subjectively, consciously.” p. 26
Metzinger, 2003	**Phenomenal self as not necessary for conscious experience:** “(…) this phenomenal quality of ‘mineness’ or bodily ‘selfhood’ is by no means a *necessary* precondition of conscious experience (…)” p. 334
Blanke and Metzinger, 2009	**Minimal phenomenal selfhood (MPS) as a phenomenal property:** “MPS is a phenomenal property, namely the conscious experience of *being a self*. It is the experience of being a distinct, holistic entity capable of global self-control and attention, possessing a body and a location in space and time” p. 7
Limanowski and Blankenburg, 2013	**Minimal (phenomenal) selfhood is underpinned by a representational structure:** “…minimal selfhood emerges as the result of pre-reflexive self-modeling, i.e., through an organism’s model of the world that is phenomenologically centered onto the self. Thereby, Metzinger’s (2010) account builds on the proposition that the brain is a representational system that needs to interpret the world (…) For this system-model to be successful, i.e., of adaptive value, ‘the self needs to be embedded into the causal network of the physical world’.” pp. 1–2
Salomon, 2017	**Minimal (phenomenal) self as a representation of being distinct from the environment:** “The reviewed studies point to the unconscious integration of multisensory signals, supported by predictive models from motor action as the basis of the minimal self. The correspondences between these exteroceptive and interoceptive sensory signals allow a fundamental representation of the organism as a discrete agent, allowing a functional segregation from the environment and conspecifics” p. 97
Christoff et al., 2011	**The self understood in a functional way, as a result of self-specifying process:**“Self specifying [process]: any process that specifies the self as subject and agent by implementing a functional self/non-self distinction” p. 104
Hohwy and Michael, 2017	**The representational structure underlying the phenomenal “Me”:** “…agents model the self as a hierarchy of hidden, endogenous causes, and further, that the self is identical to these causes (…) The self-model is a hierarchical construct whose levels are linked by message-passing as top-down predictions are generated and bottom-up prediction errors minimized.”, p. 369
Seth, 2013	**Embodied selfhood as grounded in (representations of) specific form of signals:** “emotion and embodied selfhood are grounded in active inference of those signals most likely to be ‘me’ across interoceptive and exteroceptive domains” p. 570
Zahavi and Kriegel, 2016	**Two interpretations of for-me-ness, a deflationary (implicating that there is no phenomenal “I,” only phenomenal “Me”), and a non-deflationary (implicating that for-me-ness represents the phenomenal “I”):** “The for-me-ness of experience still admits of two crucially different interpretations. According to a deflationary interpretation, it consists simply in the experience occurring in someone (a ‘me’). On this view, for-me-ness is a non-experiential aspect of mental life—a merely metaphysical fact, so to speak, not a phenomenological fact. The idea is that we ought to resist a no-ownership view according to which experiences can occur as free-floating unowned entities (…) In contrast, a non-deflationary interpretation construes for-me-ness as an experiential aspect of mental life, a bona fide phenomenal dimension of consciousness. On this view, to say that an experience is for me is precisely to say something more than that it is in me. It is to state not only a metaphysical fact, but also a phenomenological fact. (…) We favor a non-deflationary interpretation”, pp. 36–37


*The emphasis was put on theories explaining the self as a form of self-model.*

## “Me” As An Object Of Experience: Phenomenology Of Self-Consciousness

*The words* ME, *then, and* SELF, *so far as they arouse feeling and connote emotional worth, are* OBJECTIVE *designations, meaning* ALL THE THINGS *which have the power to produce in a stream of consciousness excitement of a certain particular sort* (James, 1890, p. 319, emphasis in original).

James (1890) chose the word “Me” to refer to self-as-object. What does it mean? In James’ (1890) view, it reflects “all the things” which have the power to produce “excitement of a certain particular sort.” This certain kind of excitement is nothing more than some form of experiential quality of me-ness, mine-ness, or similar - understood in a folk-theoretical way (this is an important point, because these terms have recently acquired technical meanings in philosophy, e.g., Zahavi, 2014; Guillot, 2017). What are “all the things”? The classic formulation suggests that James (1890) meant physical objects and cultural artifacts (material self), human beings (social self), and mental processes and content (spiritual self). These are all valid categories of self-as-object, however, for the purpose of this paper I will limit the scope of further discussion only to “objects” which are relevant when speaking about consciousness. Therefore, rather than speaking about, for example, my car or my body, I will discuss only their conscious representations. This limits the scope of self-as-object to one category of “things” – conscious mental content.

Let us now reformulate James’ (1890) idea in more contemporary terms and define “Me” as the totality of all content of consciousness that is experienced as self-related. Content of consciousness is meant here in a similar way to Chalmers (1996), who begins “*The conscious mind*” by providing a list of different kinds of conscious content. He delivers an extensive (without claiming that exhaustive) collection of types of experiences, which includes the following^4^: visual; auditory; tactile; olfactory; experiences of hot and cold; pain; taste; other bodily experiences coming from proprioception, vestibular sense, and interoception (e.g., headache, hunger, orgasm); mental imagery; conscious thought; emotions. Chalmers (1996) also includes several other, which, however, reflect states of consciousness and not necessarily content *per se*, such as dreams, arousal, fatigue, intoxication, and altered states of consciousness induced by psychoactive substances. What is common to all of the types of experience from the first list (conscious contents) is the fact that they are all, speaking in James’ (1890) terms, “objects” in a stream of consciousness: “all these things are objects, properly so called, to the subject that does the thinking” (p. 325).

**FIGURE 2:**
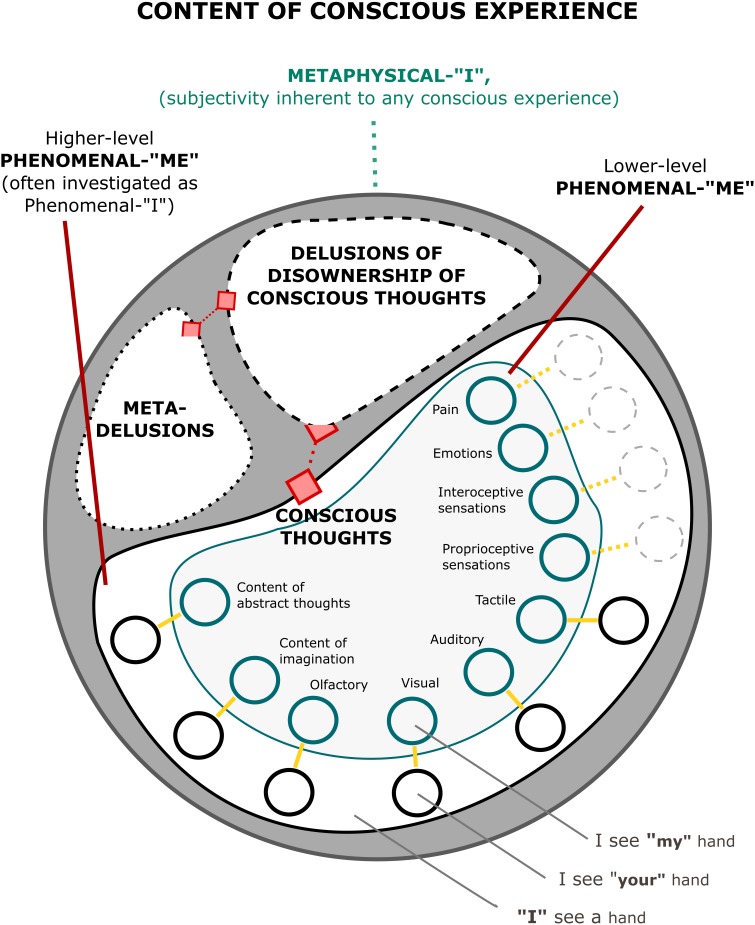
A simplified representation of a structure of phenomenal content including the metaphysical “I,” the phenomenal “Me,” and the phenomenal “I,” which can be understood (see in text) as a higher-level element of the phenomenal “Me.” Each pair of nodes connected with a yellow line represents one type of content of consciousness, with indigo nodes corresponding to self-related content, and black nodes corresponding to non-self-related content. In some cases (e.g., pain, emotions, interoceptive, and proprioceptive sensations), the black nodes are lighter and drawn with a dashed line (the same applies to links), to indicate that in normal circumstances one does not experiences these sensations as representing another person (although it is possible in thought experiments and pathologies). Multisensory/multimodal interactions have been omitted for the sake of clarity. All of the nodes compose the set of conscious thoughts, which can be formulated as “I experience X.” In normal circumstances, one does not deny ownership over these thoughts, however, in thought experiments, and in some cases of psychosis, one may experience that even such thoughts cease to feel as one’s own. This situation is represented by the shape with a dashed outline. Moreover, in special cases one can form meta-delusions, i.e., delusions about delusions – thoughts that my thoughts about other thoughts are not my thoughts (see text for description).

The self understood as “Me” can be understood as a subset of a set of all these possible experiences. This subset is characterized by self-relatedness (**Figure 2**). It can be illustrated with sensory experiences. For example, in the visual domain, I experience an image of my face as different from another person’s face. Hence, while the image of my face belongs to “Me,” the image of someone else does not (although it can be experimentally manipulated, Tsakiris, 2008; Payne et al., 2017; Woźniak et al., 2018). The same can be said about my voice and sounds caused by me (as opposed to voices of other people), and about my smell. We also experience self-touch as different from touching or being touched by a different person (Weiskrantz et al., 1971; Blakemore et al., 1998; Schutz-Bosbach et al., 2009). There is even evidence that we process our possessions differently (Kim and Johnson, 2014; Constable et al., 2018). This was anticipated by James’ (1890) notion of the material “Me,” and is typically regarded as reflecting one’s extended self (Kim and Johnson, 2014). In all of these cases, we can divide sensory experiences into the ones which do relate to the self and the ones which do not. The same can be said about the contents of thoughts and feelings, which can be either about “Me” or about something/someone else.

Characterizing self-as-object as a subset of conscious experiences specifies the building blocks of “Me” (which are contents of consciousness) and provides a guiding principle for distinguishing between self and non-self (self-relatedness). However, it is important to note two things. First, the distinction between self and non-self is often a matter of scale rather than a binary classification, and therefore self-relatedness may be better conceptualized as the strength of the relation with the self. It can be illustrated with an example of the “Inclusion of Other in Self” scale (Aron et al., 1992). This scale asks to estimate to what extent another person feels related to one’s self, by choosing among a series of pairs of more-to-less overlapping circles representing the self and another person (e.g., a partner). The degree of overlap between the chosen pair of circles represents the degree of self-relatedness. Treating self-relatedness as a matter of scale adds an additional level of complexity to the analysis, and results in speaking about the extent to which a given content of consciousness represents self, rather than whether it simply does it or not. This does not, however, change the main point of the argument that we can classify all conscious contents according to whether (or to what extent, in that case) they are self-related. For the sake of clarity, I will continue to speak using the language of binary classification, but it should be kept in mind that it is an arbitrary simplification. The second point is that this approach to “Me” allows one to flexibly discuss subcategories of the self by imposing additional constraints on the type of conscious content that is taken into account, as well as the nature of self-relatedness (e.g., whether it is ownership of, agency over, authorship, etc.). For example, by limiting ourselves to discussing conscious content representing one’s body one can speak about the bodily self, and by imposing limits to conscious experience of one’s possessions one can speak about one’s extended self.

Keeping these reservations in mind two objections can be raised to the approach to “Me” introduced here. The first one is as follows:

(1) Speaking about the self/other distinction does not make sense in regard to experiences which are always “mine,” such as prioprioception or interoception. This special status may suggest that these modalities underpin the self as “I,” i.e., the subject of experience.

This idea is present in theoretical proposals postulating that subjectivity emerges based on (representations of) sensorimotor (Gallagher, 2000; Christoff et al., 2011; Blanke et al., 2015) or interoceptive signals (Damasio, 1999; Craig, 2010; Seth et al., 2011; Park and Tallon-Baudry, 2014; Salomon, 2017). There are two answers to this objection. First, the fact that this kind of experience (this kind of content of consciousness) is always felt as “my” experience simply means that all proprioceptive, interoceptive, pain experiences, etc., are as a matter of fact parts of “Me.” They are self-related contents of consciousness and hence naturally qualify as self-as-object. Furthermore, there is no principled reason why the fact that we normally do not experience them as belonging to someone else should transform them from objects of experience (content) into a subject of experience. Their special status may cause these experiences to be perceived as more central aspects of the self than experiences in other modalities, but there is no reason to think that it should change them from something that we experience into the self as an experiencer. Second, even the special status of these sensations can be called into question. It is possible to imagine a situation in which one experiences these kinds of sensations from an organ or a body which does not belong to her or him. We can imagine that with enough training one will learn to distinguish between proprioceptive signals coming from one’s body and those coming from another person’s (or artificial) body. If this is possible, then one may develop a phenomenal distinction between “my” versus “other’s” proprioceptive and interoceptive experiences (for example), and in this case the same rules of classification into phenomenal “Me” and phenomenal “not-Me” will apply as to other sensory modalities. This scenario is not realistic at the current point of technological development, but there are clinical examples which indirectly suggest that it may be possible. For example, people who underwent transplantation of an organ sometimes experience rejection of a transplant. Importantly, patients whose organisms reject an organ also more often experience psychological rejection of that transplant (Látos et al., 2016). Moreover, there are rare cases in which patients following a successful surgery report that they perceive transplanted organs as foreign objects in themselves (Goetzmann et al., 2009). In this case, affected people report experiencing a form of disownership of the implanted organ, suggesting that they may experience interoceptive signals coming from that transplant as having a phenomenal quality of being “not-mine,” leading to similar phenomenal quality as the one postulated in the before-mentioned thought experiment. Another example of a situation in which self-relatedness of interoception may be disrupted may be found in conjoint twins. In some variants of this developmental disorder (e.g., parapagus, dicephalus, thoracopagus) brains of two separate twins share some of the internal organs (and limbs), while others are duplicated and possessed by each twin individually (Spencer, 2000; Kaufman, 2004). This provides an inverted situation to the one described in our hypothetical scenario – rather than two pieces of the same organ being “wired” to one person, the same organ (e.g., a heart, liver, stomach) is shared by two individuals. As such it may be simultaneously under control of two autonomous nervous systems. This situation raises challenging questions for theories which postulate that the root of self-as-subject lies in interoception. For example, if conjoint twins share the majority of internal organs, but possess mostly independent nervous systems, like dicephalus conjoint twins, then does it mean that they share the neural subjective frame (Park and Tallon-Baudry, 2014)? If the answer is yes, then does it mean that they share it numerically (both twins have one and the same subjective frame), or only qualitatively (their subjective frames are similar to the point of being identical, but they are distinct frames)? However, if interoception is just a part of “Me” then the answer becomes simple – the experiences can be only qualitatively identical, because they are experienced by two independent subjects.

All of these examples challenge the assumption that sensori-motor and interoceptive experiences are necessarily self-related and, as a consequence, that they can form the basis of self-as-subject. For this reason, it seems that signals coming from these modalities are more appropriate to underlie the phenomenal “Me,” for example in a form of background self-experience, or “phenomenal background” (Dainton, 2008, 2016), rather than the phenomenal “I.”

The second possible objection to the view of self-as-object described in this section is the following one:

(2) My thoughts and feelings may have different objects, but they are always my thoughts and feelings. Therefore, their object may be either “me” or “other,” but their subject is always “I.” As a consequence, even though my thoughts and feelings constitute contents of my consciousness, they underlie the phenomenal “I” and not the phenomenal “Me.”

It seems to be conceptually misguided to speak about one’s thoughts and feelings as belonging to someone else. This intuition motivated Wittgenstein (1958) to write: “there is no question of recognizing a person when I say I have toothache. To ask ‘are you sure it is you who have pains?’ “would be nonsensical” (Wittgenstein, 1958). In the Blue Book, he introduced the distinction between the use of “I” as object and as subject (see **Table 1** for a full relevant quote) and suggested that while we can be wrong about the former, making a mistake about the latter is not possible. This idea was further developed by Shoemaker (1968) who introduced an arguably conceptual truth that we are immune to error through misidentification relative to the first-person pronoun, or IEM in short. For example, when I say “I see a photo of my face in front of me” I may be mistaken about the fact that it is my face (because, e.g., it is a photo of my identical twin), but I cannot be mistaken that it is me who is looking at it. One way to read IEM is that it postulates that I can be mistaken about self-as-object, but I cannot be mistaken about self-as-subject. If this is correct then there is a radical distinction between these two types of self that provides a strong argument to individuate them. From that point, one may argue that IEM provides a decisive argument to distinguish between phenomenal “I” (self-as-subject) and phenomenal “Me” (self-as-object).

Before endorsing this conclusion, let us take a small step back. It is important to note that in the famous passage from the Blue Book Wittgenstein (1958) did not write about two distinct types of self. Instead, he wrote about two ways of using the word “I” (or “my”). As such, he was more concerned with issues in philosophy of language than philosophy of mind. Therefore, a natural question arises – to what extent does this linguistic distinction map onto a substantial distinction between two different entities (types of self)? On the face of it, it seems that there is an important difference between these two uses of self-referential words, which can be mapped onto the experience of being a self-as-subject and the experience of being a self-as-object (or, for example, the distinction between bodily ownership and thought authorship, as suggested by Liang, 2014). However, I will argue that there are reasons to believe that the phenomenal “I,” i.e., the experience of being a self-as-subject may be better conceptualized as a higher-order phenomenal “Me” – a higher-level self-as-object.

Psychiatric practice provides cases of people, typically suffering from schizophrenia, who describe experiences of dispossession of thoughts, known as delusions of thought insertion (Young, 2008; Bortolotti and Broome, 2009; Martin and Pacherie, 2013). According to the standard account, the phenomenon of thought insertion does not represent a disruption of sense of ownership over one’s thoughts, but only loss of sense of agency over them. However, the standard account has been criticized in recent years by theorists arguing that thought insertion indeed represents loss of sense of ownership (Metzinger, 2003; Billon, 2013; Guillot, 2017; López-Silva, 2017). One of the main arguments against the standard view is that it runs into serious problems when attempting to explain obsessive intrusive thoughts in clinical population and spontaneous thoughts in healthy people. In both cases, subjects report lack of agency over thoughts, although they never claim lack of ownership over them, i.e., that these are not their thoughts. However, if the standard account is correct, obsessive thoughts should be experienced as belonging to someone else. The fact that they are not suggests that something else must be disrupted in delusions of thought insertion, i.e., sense of ownership^5^ over them. If one can lose sense of ownership over one’s thoughts then it has important implications, because then one becomes capable of experiencing one’s thoughts “as someone else’s,” or at least “as not-mine.” However, when I experience my thoughts as not-mine I do it because I’ve taken a stance towards my thoughts, which treats them as an object of deliberation. In other words, I must have “objectified” them to experience that they have a quality of “feeling as if they are not mine.” Consequently, if I experience them as objects of experience, then they cannot form part of my self as subject of experience, because these two categories are mutually exclusive. Therefore, what seemed to constitute a phenomenal “I” turns out to be a part of thephenomenal “Me.”

If my thoughts do not constitute the “I” then how do they fit into the structure of “Me”? Previously, I asserted that thoughts with self-related content constitute “Me,” while thoughts with non-self related content do not. However, just now I argued in favor of the claim that all thoughts (including the ones with non-self-related content) that are experienced as “mine” belong to “Me.” How can one resolve this contradiction?

A way to address this reservation can be found in Metzinger’s (2003; 2010) self-model theory. Metzinger (2003, 2010) argues that the experience of the self can be understood as underpinned by representational self-models. These self-models, however, are embedded in the hierarchical representational structure, as illustrated by an account of ego dissolution by Letheby and Gerrans (2017):

Savage suggests that on LSD “[changes] in body ego feeling usually precede changes in mental ego feeling and sometimes are the only changes” (1955, 11), (…) This common temporal sequence, from blurring of body boundaries and loss of sense of ownership for body parts through to later loss of sense of ownership for thoughts, speaks further to the hierarchical architecture of the self-model. (Letheby and Gerrans, 2017, p. 8)

If self-models underlying the experience of self-as-object (“Me”) are hierarchical, then the apparent contradiction may be easily explained by the fact that when speaking about the content of thoughts and the thoughts themselves we are addressing self-models at two distinct levels. At the lower level we can distinguish between thoughts with self-related content and other-related content, while on the higher level we can distinguish between thoughts that feel “mine” as opposed to thoughts that are not experienced as “mine.” As a result, this thinking phenomenal “I” experienced in feeling of ownership over one’s thoughts may be conceived as just a higher-order level of Jamesian “Me.” As such, one may claim that there is no such thing as a phenomenal “I,” just multilevel phenomenal “Me.” However, an objection can be raised here. One may claim that even though a person with schizophrenic delusions experiences her thoughts as someone else’s (a demon’s or some malicious puppet master’s), she can still claim that:

Yes, “I” experience my thoughts as not mine, but as demon’s.” My thoughts feel as “not-mine,” however, it’s still me (or: “I”) who thinks of them as “not-mine.”

As such, one escapes “objectification” of “I” into “Me” by postulating a higher-level phenomenal-“I.” However, let us keep in mind that the thought written above constitutes a valid thought by itself. As such, this thought is vulnerable to the theoretical possibility that it turns into a delusion itself, once a psychotic person forms a meta-delusion (delusion about delusion). In this case, one may begin to experience that: “I” (I_1_) experience that the “fake I” (I_2_), who is a nasty pink demon, experiences my thoughts as not mine but as someone else’s (e.g., as nasty green demon’s). In this case, I may claim that the real phenomenal “I” is I_1_, since it is at the top of the hierarchy. However, one may repeat the operation of forming meta-delusions *ad infinitum* (as may happen in psychosis or drug-induced psychedelic states) effectively transforming each phenomenal “I” into another “fake-I” (and consequently making it a part of “Me”).

The possibility of meta-delusions illustrates that the phenomenal “I” understood as subjective thoughts is permanently vulnerable to the threat of losing the apparent subjective character and becoming an object of experience. As such it seems to be a poor choice for the locus of subjectivity, since it needs to be constantly “on the run” from becoming treated as an object of experience, not only in people with psychosis, but also in all psychologically healthy individuals if they decide to reflect on their thoughts. Therefore, it seems more likely that the thoughts themselves cannot constitute the subject of experience. However, even in case of meta-delusions there seems to be a stable deeper-level subjectivity, let us call it the deep “I,” which is preserved, at least until one loses consciousness. After all, a person who experiences meta-delusions would be constantly (painfully) aware of the process, and often would even report it afterwards. This deep “I” cannot be a special form of content in the stream of consciousness, because otherwise it would be vulnerable to becoming a part of “Me.” Therefore, it must be something different.

There seem to be two places where one can look for this deep “I”: in the domain of phenomenology or metaphysics. The first approach has been taken by (Zahavi and Kriegel, 2016) who argue that “all conscious states’ phenomenal character involves for-me-ness as an experiential constituent.” It means that even if we rule out everything else (e.g., bodily experiences, conscious thoughts), we are still left with some form of irreducible phenomenal self-experience. This for-me-ness is not a specific content of consciousness, but rather “refers to the distinct manner, or *how*, of experiencing” (Zahavi, 2014).

This approach, however, may seem inflationary and not satisfying (e.g., Dainton, 2016). One reason for this is that it introduces an additional phenomenal dimension, which may lead to uncomfortable consequences. For example, a question arises whether for-me-ness can ever be lost or replaced with the “*how* of experiencing” of another person. For example, can I experience my sister’s for-me-ness in my stream of consciousness? If yes, then how is for-me-ness different from any other content of consciousness? And if the answer is no, then how is it possible to distil the phenomenology of for-me-ness from the metaphysical fact that a given stream of consciousness is always experienced by this and not other subject?

An alternative approach to the problem of the deep “I” is to reject that the subject of experience, the “I,” is present in phenomenology (like Hume, 1739/2000; Prinz, 2012; Dainton, 2016), and look for it somewhere else, in the domain of metaphysics. Although James (1890) did not explicitly formulate the distinction between “Me” and “I” as the distinction between the phenomenal and the metaphysical self, he hinted at it at several points, for example when he concluded the Chapter on the self with the following fragment: “(...) a postulate, an assertion that there *must be a knower* correlative to all this *known*; and the problem *who that knower is* would have become a metaphysical problem” (James, 1890, p. 401).

## “I” As A Subject Of Experience: Metaphysics Of Subjectivity

Thoughts which we actually know to exist do not fly about loose, but seem each to belong to some one thinker and not to another (James, 1890, pp. 330–331).

Let us assume that phenomenal consciousness exists in nature, and that it is a part of the reality we live in. The problem of “I” emerges once we realize that one of the fundamental characteristics of phenomenal consciousness is that it is always subjective, that there always seems to be some subject of experience. It seems mistaken to conceive of consciousness which do “fly about loose,” devoid of subjective character, devoid of being someone’s or something’s consciousness. Moreover, it seems that subjectivity may be one of the fundamental inherent properties of conscious experience (similar notions can be found in: Berkeley, 1713/2012; Strawson, 2003; Searle, 2005; Dainton, 2016). It seems highly unlikely, if not self-contradictory, that there exists something like an objective conscious experience of “what it is like to be a bat” (Nagel, 1974), which is not subjective in any way. This leads to the metaphysical problem of the self: why is all conscious experience subjective, and what or who is the subject of this experience? Let us call it the problem of the metaphysical “I,” as contrasted with the problem of the phenomenal “I” (i.e., is there a distinctive experience of being a self as a subject of experience, and if so, then what is this experience?), which we discussed so far.

The existence of the metaphysical “I” does not entail the existence of the phenomenal self. It is possible to imagine a creature that possesses a metaphysical “I,” but does not possess any sense of self. In such a case, the creature would possess consciousness, although it would not experience anything as “me,” nor entertain any thoughts/feelings, etc., as “I.” In other words, it is a possibility that one may not experience self-related content of consciousness, while being a sentient being. One example of such situation may be the experience of a dreamless sleep, which “is characterized by a dissolution of subject-object duality, or (…) by a breakdown of even the most basic form of the self-other distinction” (Windt, 2015). This is a situation which can be regarded as an instance of the state of minimal phenomenal experience – the simplest form of conscious experience possible (Windt, 2015; Metzinger, 2018), in which there is no place for even the most rudimentary form of “Me.” Another example may be the phenomenology of systems with grid-like architectures which, according to the integrated information theory (IIT, Tononi et al., 2016), possess conscious experience^6^. If IIT is correct, then these systems experience some form of conscious states, which most likely lack any phenomenal distinction between “Me” and “not-Me.” However, because they may possess a stream of conscious experience, and conscious experience is necessarily subjective, there remains a valid question: who or what is the subject of that experience?

The question of what exactly is the metaphysical subject of experience can have different answers. There has been a long history of theories of the self (Barresi and Martin, 2011) and some of them directly address this issue. Platonic or Cartesian notions of the soul are good examples of an approach providing one answer to this question: conscious experience is subjective, because there exists a non-material being (self, soul) which is the subject of this experience (see **Table 3**). Other solutions tend to either define the self in less metaphysically expensive ways (Johnston, 1987; Strawson, 2000; Dainton, 2008), define it as a formal feature of consciousness (Searle, 2005), or deny the need to postulate its existence (Metzinger, 2003). What is crucial here, however, is that the problem of the metaphysical self is a different issue and requires a different methodology, than the problem of the phenomenal self.

**Table 3:** Examples of theories of the self-as-subject (“I”) in the context of consciousness, as theories of the metaphysical self, with representative quotes illustrating each position.

	Description and illustrative quote
**Metaphysical Self**	**The self as responsible (or not) for the subjectivity inherent to all conscious experience**
Descartes, 1637/2006	**Metaphysical “I” as an immaterial soul, which can exist independently of the body:** “I thereby concluded that I was a substance whose whole essence or nature resides only in thinking, and which, in order to exist, has no need of place and is not dependent on any material thing. Accordingly this ‘I,’ that is to say, the Soul by which I am what I am, is entirely distinct from the body and is even easier to know than the body; and would not stop being everything it is, even if the body were not to exist.”, p. 29
Johnston, 1987	**Metaphysical “I” as a *bare-locus* of consciousness:** “we are what I will call *bare loci* of mental life, that is, possessors of mental life whose survival requires no amount of either bodily or mental continuity,” p. 70
Strawson, 2003	**Metaphysical “I” as a thin subject:** “there cannot be a subject of experience, at any given time, unless some experience exists for it to be a subject of, at that time. (…) the thin conception according to which a subject of experience is an inner thing of some sort that does not and cannot exist at any given time unless it is having experience at that time.”, p. 284
Dainton, 2016	**The self as a capacity for experience, which underpins the metaphysical “I”:** “If all that is essential to the nature of a subject is the capacity to have experiences, a natural next step is to hold that a subject simply is a capacity for experience. In the case of very simple or primitive subjects (a simple worm-like creature, for example), this capacity might very well be very simple too: perhaps there are subjects who are capable of only a single kind of experience (e.g., a sensation of warmth). The stream of consciousness of such a subject will take the form of a continuous flow of a single kind of sensation.”, p. 116
Searle, 2005	**The self (metaphysical “I”) as a formal feature of consciousness:** “The x in question is the self in at least one sense of the word. Notice that the postulation of the self is not the postulation of a separate entity distinct from the conscious field but rather it is a formal feature of the conscious field.”, p. 15
Metzinger, 2010	**The eliminativist position on the metaphysical “I”:** “One of the ontological claims of this theory is that the self is not a substance in the technical philosophical sense of – ontological self-subsistence – of something that could maintain its existence on its own, even if the body, the brain, or everything else disappeared. It is not an individual entity or a mysterious thing in the metaphysical sense. No such things as selves exist in the world: Selves and subjects are not part of the irreducible constituents of reality”, p. 26


What sort of methodology, then, is appropriate for investigating the metaphysical self? It seems that the most relevant methods come from the toolbox of metaphysics. This toolbox includes classical philosophical methods such as thought experiments and logical analysis. However, methodology of metaphysics is an area of open discussion, and at present there are no signs of general consensus. One of the most debated issues in this field, which is especially relevant here, is to what extent the methodology of metaphysics is continuous with the methodology of natural sciences (see Tahko, 2015, Chapter 9 for an overview). The positions span the spectrum between the claim that science and metaphysics are fully autonomous on the one side and the claim that metaphysics can be fully naturalized on the other. Discussing this issue goes way beyond the scope of this paper. However, if these two areas are at least to some extent related (i.e., not fully autonomous), then one may argue that scientific methods can be at least of some relevance in metaphysics and consequently for investigations of the metaphysical “I.”

One example in which empirical results seem to be able to influence theoretical investigations of the metaphysical self is through imposing constraints on philosophical theories. For example, because the metaphysical self is inherently related to consciousness, we should expect that different theories of consciousness should place different constraints on what a metaphysical self can be. Then, if one theory of consciousness acquires stronger empirical support than the others, we can also treat this as evidence for the constraints on the self that this theory implies.

Let us look at an example of IIT to illustrate this point. According to IIT (Oizumi et al., 2014; Tononi et al., 2016) the content of conscious experience is defined by the so-called informational “complex” which is characterized by maximally integrated information (which can be measured by calculating the value of Φ^max^). This complex then defines the stream of conscious experience. However, what happens if there is more than one such complex in one person? In this case, as Tononi et al. (2016) wrote:

According to IIT, two or more non-overlapping complexes may coexist as discrete physical substrates of consciousness (PSCs) within a single brain, each with its own definite borders and value of Φ^max^. The complex that specifies a person’s day to day stream of consciousness should have the highest value of Φ^max^ – that is, it should be the “major” complex. In some conditions, for example, after a split-brain operation, the major complex may split. In such instances, one consciousness, supported by a complex in the dominant hemisphere and with privileged access to Broca’s area, would be able to speak about the experience, but would remain unaware of the presence of another consciousness, supported by a complex in the other hemisphere, which can be revealed by carefully designed experiments. (Tononi et al., 2016, p. 455)

This fragment suggests that in IIT the metaphysical “I” can be understood as tied to a complex of maximally integrated information. In this case, a split-brain patient would possess two metaphysical selves, because as a consequence of an operation her or his brain hosts two such complexes. On the face of it, it seems to be a plausible situation (*cf.* Bayne, 2010). However, in the sentence which immediately follows, Tononi et al. (2016) suggest that:

An intriguing possibility is that splitting of the PSC may also occur in healthy people during long-lasting dual-task conditions – for example, when driving in an auto-pilot like manner on a familiar road while listening to an engaging conversation (Tononi et al., 2016, p. 455)

The implications of this possibility are much more severe, because it postulates that in a matter of minutes or seconds a complex can dynamically divide into several complexes, and individual complexes can merge into one major complex. How do the complexes understood in this way then relate to the metaphysical “I”? Unfortunately, IIT is silent about this issue, but there seem to be at least two responses to this question. First, one may argue that the self does not need to be limited to one complex, but that the same metaphysical “I” can be present in all of the simultaneous streams of consciousness (complexes). However, this solution is at odds with both common-sense intuition and IIT itself. It would presuppose not only an extremely disunified view of consciousness, but even lead to self-contradictory consequences. The metaphysical “I” can be thought of as the metaphysical fact that any given stream of consciousness is subjectively experienced by some “self” (regardless of what that self might be). However, in a disunified view of an organism’s consciousness this metaphysical “I” would at the same time a) be the subject of experience of all of the complexes within this organism, and b) be the subject of experience of only one of these complexes while being blind to the others (as claimed by IIT: two complexes are not “co-conscious” with each other). It presents a contradiction and strongly suggests that the metaphysical “I” cannot be underpinned by multiple independent complexes. It leaves us with the second option, which is to bite the bullet and accept that IIT implies that the metaphysical “I” persists either as long as a given complex, or for an even shorter period of time, for example for just up to a few seconds, as suggested by Strawson (2000, 2010). It means that if IIT (and the analysis outlined above) is correct then the metaphysical “I” turns out to be radically different from our intuitive understanding of subject-of-experience as persisting continuously life-long stream of consciousness. However, if empirical evidence in support of the current version of IIT becomes strong enough, it may suggest that our common-sense intuitions about self-as-subject may be mistaken. On the other hand, different theories of phenomenal consciousness (and even different versions of IIT) may imply different constraints on the metaphysical “I,” and the extent to which they are supported by empirical evidence may suggest a way to say something about what the subject of conscious experience is.

Overall, assuming that metaphysics is not fully independent from science, the relevant methodology for investigating the metaphysical “I” is a combination of toolboxes of metaphysics and empirical science. This contrasts with the phenomenal “Me,” where the relevant toolkit includes methods from phenomenology and science. The second point, which has been illustrated with an example of IIT, is that it is important to explicitly spell out the implications of different theories of consciousness in regard to what is the subject of conscious experience, as it may provide the best way forward towards solving this issue.

## Understanding Predictive Coding Theories Of The Self

Recently, there has been a huge number of attempts to explain the self through the framework of predictive coding (PC) and the free energy principle (FEP). In this final section of the paper, I will use PC theories of the self as a working example demonstrating practical consequences of implementing the Jamesian distinction between “Me” and “I.” I will suggest that PC theories of the self target different dimensions of self-as-object, understood as a hierarchical structure of self-models (Metzinger, 2003, 2010), and as such provide a valuable framework to understand the self. However, I will also explain why PC and the FEP do not allow us to say much about self-as-subject (the metaphysical “I”).

According to PC, the brain can be understood as an inference machine which hosts and continuously updates a probabilistic model of the world, which it uses to infer hidden causes behind the sensory data (for a more detailed introduction see: Friston et al., 2006; Friston, 2009, 2010; Friston and Kiebel, 2009; Hohwy, 2013; Clark, 2016). It accomplishes this by continuously issuing predictions and comparing them with sensory data, with the discrepancy between predictions and data being propagated further up the hierarchy as prediction errors. As such, PC postulates that the brain can be seen as a hierarchical structure of generative models (which are responsible for issuing predictions). Prediction errors which arise at lower levels serve as data to be compared with predictions at the higher levels. This view of the mind inverts the classical feedforward view in which perception is a predominantly bottom-up process. In PC, instead, perception is mostly driven by top-down predictions, with bottom-up prediction errors serving the function of feedback helping to choose model with the most explanatory power. Moreover, in an extension of PC, which is known as active inference, action is also understood as a way of maximizing the fit of one’s internal models to reality. The main idea behind active inference is that rather than changing the model in order to better fit the data, one can act on the world and change it according to predictions issued by the currently dominating model. As a consequence, the whole perception-action cycle can be understood as driven by one overarching goal, i.e., long-term minimization of prediction errors.

The FEP is a further generalization of PC. It postulates that all living organisms operate under the principle to minimize the so-called “variational free energy,” which is an information theoretical measure which roughly can be understood as a measure of uncertainty (Friston et al., 2006; Friston, 2009). One of the main claims of this theory is that organisms which act according to FEP (i.e., they act in a way to minimize free energy in the long term) will, in effect, implicitly approximate Bayesian inference. It means that they will combine their prior knowledge (represented by their model of the world) with the incoming sensory input in a mathematically optimal way.

Both PC and the FEP have recently gained huge popularity and motivated a number of theories attempting to explain various aspects of cognition within this framework. It includes numerous attempts to understand different facets of the self, such as sense of bodily ownership (Apps and Tsakiris, 2014), sense of self in agency and perception (Hohwy, 2007), the influence of interoception on self-consciousness (Seth et al., 2011; Seth, 2013), social aspects of the self (Moutoussis et al., 2014; Friston and Frith, 2015), the relationship with minimal phenomenal selfhood (Limanowski and Blankenburg, 2013), and even psychodynamical interpretations of the self (Carhart-Harris and Friston, 2010; Fotopoulou, 2012). The most comprehensive treatment of the self from the PC perspective (Hohwy and Michael, 2017) also exemplifies most of the crucial points made by other PC theories of the self. At the beginning of their paper Hohwy and Michael (2017) describe the self in the following words:

We use a general computational framework for brain function to develop a theory of the self. The theory is that the self is an inferred model of endogenous, deeply hidden causes of behavior. (…) we discuss why such a set of hidden endogenous causes should qualify as a self. (Hohwy and Michael, 2017, p. 363)

The self, as seen from this perspective, is essentially a hierarchical model of endogenous hidden causes of sensory input. Or, in more classical terms, it can be said that it is a hierarchical representational structure (*cf.* Clark, 2016; Williams, 2017) which allows one to distinguish between endogenous causes (what is caused by me) and exogenous causes (what is caused by something else). This distinction can be illustrated with an example of a comparison between seeing a movement of my virtual hand and of a virtual hand of someone else. If adequately prepared, in both cases the image of a hand and its movement may be identical. However, in one case I can realize that the movement of the hand is congruent with my intentions (manifested through my actions performed using a computer controller) and, as a consequence, infer that the cause of the hand’s movement is me. On the other hand, I may fail to notice any congruence between my intentions and the movement and hence infer that the hidden cause behind the movement I observe is some other person. According to Hohwy and Michael (2017), the self is just a set of such hidden endogenous causes. Although not necessarily in full agreement with this picture in regard to the details, all other PC theories of the self listed above also speak about the self as underpinned by hierarchy of generative models, which are preoccupied with conducting probabilistic inference aimed to infer hidden causes of observed data patterns. This inference is then postulated to underlie specific types of conscious self-experience, i.e., different facets of the sense of self.

As such, one common theme among all PC theories of the self is the following: aspects of conscious experience of the self are underpinned by a representational structure in the form of hierarchical generative models. In its core, it is the same idea as the one introduced earlier by Metzinger (2003, 2010), i.e., that our phenomenal experience of the self is underpinned by a representational structure of unconscious self-models (see also: Crane, 2003; Chalmers, 2004, for a discussion about the relationship between representational and conscious content). Once an unconscious self-model enters conscious awareness, it generates a corresponding self-related conscious content (see: Metzinger, 2006, 2014, for an explicit distinction between the levels of representations and conscious content in regard to the bodily self). The same mechanism is at work in PC theories – the dynamic process of model selection leads to suppression of some models but allows other models to enter awareness in the form of conscious content. This mechanism allows PC to explain self-related content of consciousness, which is essentially nothing else than the James’ (1890) self-as-object of experience. This is how PC and the FEP help to understand the phenomenal “Me” – by describing the structure and dynamics of the underlying representational architecture.

To what extent PC and FEP can provide us with any help when confronted with the task to explain the metaphysical “I”? Here, I will argue that in contrast to the phenomenal “Me,” the issues pertaining to the metaphysical “I” are outside of its reach. The reason for this is a consequence of the fact that PC is in principle agnostic in regard to the issue of what brings representational content into the scope of conscious experience. In general, this can be regarded as an advantage, because this way PC accounts of self-experience can avoid the burden of being hostage to any specific theory of consciousness, and stay in principle compatible with most of them (e.g., see Hohwy, 2013, Chapter 10 for an attempt to combine PC with ideas from Global Neuronal Workspace theory: Dehaene and Changeux, 2011; Dehaene, 2014). However, it also makes PC fundamentally underspecified when treated as a theory which is used to explain issues related to consciousness. While, as suggested before, PC is a valuable framework to describe the representational structure underlying conscious content, it runs into problems when used to explain why certain content is conscious in the first place. One way in which PC and FEP can attempt to retain relevance is by aiming to explain access consciousness (Block, 1995) – a functional mechanism which allows that “some of the attended information eventually enters our awareness and becomes reportable to others” (Dehaene, 2014). However, the problem of the metaphysical “I” becomes a relevant issue only when approached in the context of phenomenal consciousness – the type of consciousness which is loaded with the burden of the so-called “hard problem” (Chalmers, 1996).

This is where PS and FEP encounter a dead end, as the problem enters the area which belongs more to metaphysics than empirical science (at least in the light of the current state of affairs). In order to provide an account of the metaphysical self, one needs to begin with at least some form of a theory of phenomenal consciousness and its place in physical reality. At present FEP (and PC) does not provide such a theory. Recently, Friston (2018) suggested that FEP can be used to understand consciousness, although the fact that he discusses consciousness in functionalist terms (consciousness is related to counterfactual inference^7^) suggests that his proposal aims to explain access consciousness, making it irrelevant for the problem of metaphysical “I.”

To summarize, the fact that PC and the FEP are not theories of phenomenal consciousness, and seem not to impose any constraints on these theories, has important consequences for what type of self they can explain. As I argued, they have the potential to substantially contribute to the issue of different levels of the phenomenal “Me” (self-as-object) by describing the structure and dynamics of the level of representational content, which are reflected at the level of conscious experience. However, they are not suited to explain the metaphysical “I” (self-as-subject) because they do not address the issue of the place of consciousness in nature. Hence, the main claim is that while PC can be seen as a useful framework to investigate phenomenology of “Me,” it is in principle unsuitable to provide answers to questions about the metaphysics of “I.”

## Conclusion

I placed the debate of the self in the domain of consciousness (as opposed to the self understood as e.g., a representational structure, a physical object, or a spiritual entity) and argued that (1) conceptually, the distinction between “Me” and “I” may reflect the distinction between theoretical problems of the phenomenal self and the metaphysical self, respectively (although the notion of for-me-ness may complicate this picture), and (2) that what is described in the literature as the phenomenal “I” can be regarded as just a higher-level part of the phenomenal “Me” [which can be understood as Metzinger’s (2018) phenomenal self-model].

The first claim draws attention to the distinction between “I” and “Me,” which suggests that these two theoretical issues should be investigated independently, using two different methodologies. While “Me” can be investigated using phenomenology and scientific methodology, “I” is typically a metaphysical problem (perhaps with the exception of non-deflationary understandings of for-me-ness) and it is arguable to what extent it can be approached using standard scientific methods. Therefore, it is important to clearly state which problem one approaches when discussing the self in the context of consciousness (see **Tables 2**, **3** for some examples).

The second claim, the postulate to treat what is usually described as phenomenal “I” as just a part of the phenomenal “Me,” has two implications. The first is constructive. Investigating issues which are typically regarded in cognitive science as “I” from the perspective of “Me” may contribute towards better understanding of self-consciousness by emphasizing that these two research areas may have much more in common than it appears. Rather than using two distinct terms, which suggest that we are dealing with two fundamentally different problems, we may approach them as just two facets of the same multidimensional research problem. One such approach is to treat both of them as just different levels in the hierarchical structure of the phenomenal self-model (Metzinger, 2003, 2009, 2010), an approach which can be (and implicitly is) shared by recent theories of the self, especially within the framework of PC.

The second implication is pragmatic. Refraining from using the term “I” when speaking in the context of phenomenology and using it only in the metaphysical context may reduce conceptual confusion in regard to this term. However, it will also mean forfeiting an important distinction (“Me” versus “I”) which has already gained traction in cognitive science. As such, the choice to eliminate the term “I” in the context of phenomenology is a repelling option, but may be beneficial in the long term. Alternatively, one may use more specific terms, such as “sense of ownership over an experience” to reflect what is meant by “I” in the Wittgensteinian tradition, or, e.g., “sense of ownership of interoceptive signals” when discussing the role of interoception. A second option may be to recast the distinction used in cognitive science in different terms. One proposal is to explicitly speak about it as the distinction between the experience/sense of “Me” versus the experience/sense of “I” (rather than just “Me” and “I”). The task here would be, however, to prove that there is a qualitative difference between them, and to demarcate the exact border.

## Author Contributions

The article has been solely the work of MW.

## Conflict of Interest Statement

The author declares that the research was conducted in the absence of any commercial or financial relationships that could be construed as a potential conflict of interest.

## Footnotes

1 Therefore, whenever I use the term “I" I mean self-as-subject (of experience), and whenever I use the term “Me" I mean self-as-object (of experience). This assumption reflects James’ (1890) understanding of these terms (see **Table 1**). I also assume, following James (1890), that these two categories are mutually exclusive, i.e., if something is an object of experience then it cannot simultaneously be a subject of experience, and vice versa.

2 Wittgenstein (1958) himself did not discuss the issue of phenomenology of the self. However, his approach to the distinction between the use of “I” as subject and “I” as object can be seen as a starting point for contemporary discussions of phenomenology of the self-as-subject.

3 Whenever I use the prefix “phenomenal” I mean “the conscious experience of.” For example, when I write phenomenal “I”, I mean: the conscious experience of self as subject of experience (“I”). In a similar fashion I use the prefix “metaphysical” when I mean “the metaphysical entity of.”

4 Chalmers (1996) also lists “sense of self,” although it is highly controversial whether it can be treated as a distinctive type of conscious content.

5 Sometimes referred to as sense of authorship.

6 “IIT allows for certain simple systems such as grid-like architectures, similar to topographically organized areas in the human posterior cortex, to be highly conscious even when not engaging in any intelligent behavior” (Tononi et al., 2016, p. 460).

7 For example, he writes: “So where does consciousness emerge? The proposal offered here is that conscious processing has a temporal thickness or depth, which underwrites inferences about the consequences of action. This necessarily lends inference a purposeful and self-evidencing aspect that has the hallmarks of consciousness” (Friston, 2018, p. 1).
